# Identification of Endogenous Kinase Substrates by Proximity Labeling Combined with Kinase Perturbation and Phosphorylation Motifs

**DOI:** 10.1016/j.mcpro.2021.100119

**Published:** 2021-06-27

**Authors:** Tomoya Niinae, Koshi Imami, Naoyuki Sugiyama, Yasushi Ishihama

**Affiliations:** 1 Department of Molecular & Cellular BioAnalysis, Graduate School of Pharmaceutical Sciences, Kyoto University, Sakyo-ku, Kyoto, Japan; 2 PRESTO, Japan Science and Technology Agency (JST), Chiyoda-ku, Tokyo, Japan; 3 Laboratory of Clinical and Analytical Chemistry, National Institute of Biomedical Innovation, Health and Nutrition, Ibaraki, Osaka, Japan

**Keywords:** kinase substrate, BioID (proximity-dependent biotin identification), phosphoproteomics, ACN, acetonitrile, AP-MS, affinity-purification mass spectrometry, BioID, proximity-dependent biotin identification, BirA, biotin ligase, CK2, casein kinase 2, GO, gene ontology, HAMMOC, hydroxy-acid-modified metal oxide chromatography, LC/MS/MS, liquid chromatography/tandem mass spectrometry, PKA, protein kinase A, PPI, protein–protein interaction, PSM, peptide spectrum match, PWMs, position weight matrices, TMT, tandem Mass Tag

## Abstract

Mass-spectrometry-based phosphoproteomics can identify more than 10,000 phosphorylated sites in a single experiment. But, despite the fact that enormous phosphosite information has been accumulated in public repositories, protein kinase–substrate relationships remain largely unknown. Here, we describe a method to identify endogenous substrates of kinases by using a combination of a proximity-dependent biotin identification method, called BioID, with two other independent methods, kinase-perturbed phosphoproteomics and phosphorylation motif matching. For proof of concept, this approach was applied to casein kinase 2 (CK2) and protein kinase A (PKA), and we identified 24 and 35 putative substrates, respectively. We also show that known cancer-associated missense mutations near phosphosites of substrates affect phosphorylation by CK2 or PKA and thus might alter downstream signaling in cancer cells bearing these mutations. This approach extends our ability to probe physiological kinase–substrate networks by providing new methodology for large-scale identification of endogenous substrates of kinases.

## Highlights

• Identification of novel kinase interactors by BioID.
• Applying two orthogonal filters, kinase perturbation and phosphorylation motif.
• Identification of novel CK2 and PKA substrates.
• A universal method for the identification of endogenous substrates for all kinases.

## Graphical Abstract

**Figure:**
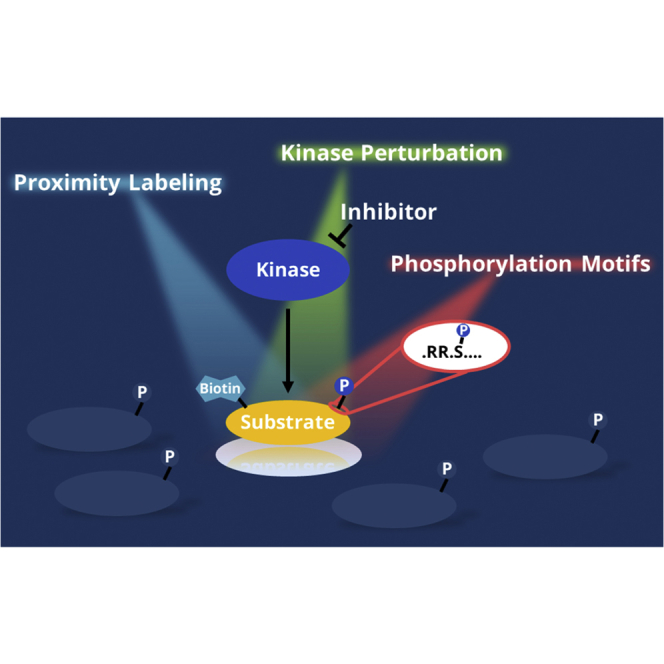

## In Brief

A method to identify endogenous substrates of kinases was described by using a combination of a BioID proximity labeling, with two other independent methods, kinase-perturbed phosphoproteomics, and phosphorylation motif matching. This approach identified 24 and 35 putative CK2 and PKA substrates, respectively.

Protein phosphorylation plays a key role in intracellular signal transduction and regulates various biological processes, including cell proliferation and differentiation. Mass spectrometry (MS)-based phosphoproteomics has made it possible to identify thousands of phosphorylated sites in single experiments (1, 2, 3). However, despite the fact that enormous phosphosite information has been accumulated in public repositories (4, 5, 6, 7), protein kinase–substrate relationships remain largely unknown both *in vitro* and *in vivo* (8).

*In vitro* kinase assay is one of the most widely used approaches to identify kinase substrates (9, 10, 11, 12, 13). We recently reported a total of 198,536 substrates for 385 kinases using *in vitro* kinase reaction with protein extracted from human cells, followed by phosphopeptide enrichment and liquid chromatography–tandem mass spectrometry (LC/MS/MS) analyses (14, 15). While this method successfully identified *in vitro* substrates and uncovered a variety of consensus motifs for kinase substrates, the identity of the endogenous kinase substrates remains enigmatic, as the physiological conditions within cells were not considered in these studies. Perturbation of kinase activity in living cells through drug treatment (16, 17) or knocking down/out a specific kinase (18, 19) allows us to monitor consequent changes in the phosphorylation level *in vivo*. However, these approaches would also indirectly affect downstream kinases, which would interfere with the identification of direct substrates (20). To overcome these issues, we and others have employed *in vitro* substrate or sequence motif information, in addition to conducting kinase-perturbed phosphoproteomics using living cells. These approaches have identified endogenous substrate candidates of protein kinase A (PKA), spleen tyrosine kinase (Syk), and Abelson tyrosine kinase (ABL) (21, 22, 23).

Although the combined use of *in vitro* substrate information and kinase-perturbed phosphoproteomic profiling has identified putative substrates in some cases, this strategy cannot distinguish substrates of downstream kinases that phosphorylate motifs similar to the target kinase. Therefore, we reasoned that an additional layer of information on kinase–protein interactions including transient and unstable interactors should allow more confident identification of endogenous substrates. Indeed, computational analyses have demonstrated that using protein–protein interaction-derived networks and kinase-specific phosphorylation sequence motifs improves the prediction of the substrate specificity (24, 25, 26). However, those studies relied on public protein interaction databases in which protein interactions were measured using conventional approaches such as affinity purification–mass spectrometry (AP-MS) and yeast two-hybrid studies. In general, kinase–substrate interactions are transient and unstable, and conventional methods fail to capture the kinase–substrate complex. To overcome this problem, AP-MS was performed in the absence of ATP and Mg^2+^ to stabilize the complex, followed by elution with buffer containing ATP and Mg^2+^ to dissociate the complex (27). Although this approach can identify *in vitro* kinase interactors, it is still difficult to apply AP-MS to living cells to identify endogenous substrates of a target kinase.

Recently, proximity labeling approaches, such as proximity-dependent biotin identification (BioID) (28) and an engineered ascorbate peroxidase (APEX) (29), have been developed to capture transient and unstable interactions, including kinase–substrate interactions (30, 31, 32). BioID is based on biotinylation of proteins proximal (~10 nm) to a mutant biotin ligase (BirA∗)-fused protein of interest; the biotinylated proteins are then captured and identified by means of streptavidin pull-down and LC/MS/MS. Thus, BioID is suitable for interactome analysis to globally capture transient and unstable interactions such as kinase–substrate interactions in cells, although it should be borne in mind that BioID captures not only transient and unstable interactors but also proximal proteins.

The aim of the present work is to establish a generic workflow for the systematic analysis of endogenous kinase–substrate relationships. For this purpose, we combined BioID-based kinase interactome analysis to capture endogenous substrates with two other independent analyses, *i.e.*, kinase-perturbed phosphoproteome analysis and phosphorylation motif analysis, in order to discriminate substrates from nonsubstrate proximal proteins.

## Experimental procedures

### Cell Culture

HEK293T cells were provided by RIKEN BRC through the National BioResource Project of the MEXT/AMED, Japan. HEK293T cells and HeLa cells (HSRRB) were cultured in Dulbecco's Modified Eagle Medium (Fujifilm Wako) containing 10% fetal bovine serum (Thermo Fisher Scientific) and 100 μg/ml penicillin/streptomycin (Fujifilm Wako).

### Cloning of BirA∗-kinase Expression Vectors

pDEST-pcDNA5-BirA∗-FLAG N-term and pDEST-pcDNA5-BirA-FLAG-GFP were a gift from Dr Anne-Claude Gingras (Lunenfeld-Tanenbaum Research Institute at Mount Sinai Hospital). The entry clones pENTR221 CK2A1 and pENTR221 PRKACA were purchased from DNAFORM and RIKEN BRC through the National BioResource Project of the MEXT/AMED, Japan, respectively. CK2A1 or PRKACA coding sequences were cloned into the destination vector pDEST-pcDNA5-BirA∗-FLAG N-term with LR clonase 2 (Thermo Fisher Scientific) using the Gateway system. Sequences of all constructs were confirmed by the Sanger method (Genewiz).

### BioID

For kinase interactome experiments, HEK293T cells in a 10 cm dish were transfected with 40 μl 1.0 mg/ml polyethylenimine (Polysciences) and 15 μg plasmid and incubated for 24 h. As the negative control, HEK293T cells in a 10 cm dish were transfected with 40 μl 1.0 mg/ml polyethylenimine for 24 h. All experiments were performed in triplicate. The cells were then incubated for 24 h in culture medium containing 50 μM biotin (Fujifilm Wako), washed, and harvested with ice-cold PBS.

### Western Blot

HEK293T cells were lysed with RIPA buffer. Supernatants after centrifugation (16,000*g*, 30 min, 4 °C) were resuspended in LiDS loading sample buffer (Thermo Fisher Scientific) containing 2-mercaptethanol. The protein samples were loaded onto a 4–12% gradient SDS–polyacrylamide gel (Thermo Fisher Scientific) and separated using electrophoresis. The proteins were then transferred to a PVDF membrane (Merck Millipore) using a semidry western blot transfer system set to a constant current of 200 mA for 30 min and stained with Ponceau S (Beacle). The membranes were blocked by incubation in 5% (w/v) BSA in Tris-buffered saline and 0.1% Tween (TBS-Tween) and then incubated for 1 h or overnight. Membranes were washed three times in TBS-Tween and developed with ECL reagent (Thermo Fisher Scientific). The primary antibodies for Flag tag, PKA, CK2 were purchased from Cell Signaling Technology. The HRP-conjugated streptavidin was purchased from Thermo Fisher Scientific.

### Drug Treatment

HEK293T cells were treated with dimethylsulfoxide, 10 μM CX-4945 (ApexBio), or 50 μM forskolin (Fujifilm Wako) for 1 h. Biological triplicates were performed.

### Sample Preparation for Biotinylated Protein Identification

Cells were washed and harvested with ice-cold PBS. The proteins were extracted into RIPA buffer (50 mM Tris-HCl [pH 7.2], 150 mM, NaCl, 1% NP-40, 1 mM EDTA, 1 mM EGTA, 0.1% SDS, protease inhibitors cocktail [Sigma-Aldrich], and 1% sodium deoxycholate), and rotated with 300 μg of streptavidin magnetic beads (Thermo Fisher Scientific) for 3 h at 4 °C. After incubation, the beads were washed with RIPA buffer three times and 50 mM ammonium bicarbonate buffer three times, then suspended in 200 μl of 50 mM ammonium bicarbonate buffer. The captured proteins were reduced with 10 mM DTT for 30 min, alkylated with 50 mM iodoacetamide for 30 min in the dark, and digested with Lys-C (Fujifilm Wako) (w/w 1:100) for 3 h, followed by trypsin (Promega) digestion (w/w 1:100) overnight at 37 °C, on the beads. The peptides were desalted using StageTip (33) with SDB-XC Empore disk membranes (GL Sciences) and suspended in the loading buffer (0.5% TFA and 4% acetonitrile [ACN]) for subsequent LC/MS/MS analyses.

### Sample Preparation for Phosphoproteome Analysis

Cells were washed and harvested with ice-cold PBS. The proteins were extracted with phase-transfer surfactant (34) using a lysis buffer (12 mM sodium deoxycholate [Fujifilm Wako], 12 mM sodium N-lauroylsarcosinate [Fujifilm Wako], 100 mM Tris-HCl [pH 9.0], containing protein phosphatase inhibitor cocktail 1 and 2 [Sigma-Aldrich], and protease inhibitors [Sigma-Aldrich]). Protein amount was determined with a BCA protein assay kit, and the proteins were reduced with 10 mM DTT for 30 min and then alkylated with 50 mM iodoacetamide for 30 min in the dark. After reduction and alkylation, proteins were digested with Lys-C (w/w 1:100) for 3 h, followed by trypsin digestion (w/w 1:100) overnight at 37 °C. Then, the peptides were desalted using SDB-XC StageTip.

Phosphopeptides were enriched from 100 μg of tryptic peptides by means of TiO_2_-based hydroxy-acid-modified metal oxide chromatography (HAMMOC) (35) and eluted with 0.5% piperidine. Phosphopeptides were labeled with tandem mass tag (TMT) (Thermo Fisher Scientific), desalted using SDB-XC StageTips, fractionated at basic pH (33), and suspended in the loading buffer (0.5% TFA and 4% ACN) for subsequent LC/MS/MS analyses.

### Immunoprecipitations

Cells were washed and harvested with ice-cold PBS. Proteins were extracted from HEK293T cells with a lysis buffer (1% NP-40, 150 mM NaCl, 25 mM Tris-HCl, pH 7.5), containing protein phosphatase inhibitor cocktail 1, 2 (Sigma-Aldrich) and protease inhibitors (Sigma-Aldrich). Cell lysates were incubated with anti-FLAG M2 magnetic beads (Sigma-Aldrich) for 1 h.

### *In vitro* Kinase Assay Using Synthetic Peptides

A mixture of synthetic peptides (SynPeptide) (10 pmol each) was reacted with 0.5 μg of each recombinant kinase (Carna Biosciences) or immunoprecipitates in 100 μl of kinase reaction buffer (40 mM Tris-HCl pH 7.5, 20 mM MgCl_2_, 1 mM ATP) at 37 °C for 3 h. The reaction was quenched by adding 10 μl 10% TFA. Then, the peptides were desalted using SDB-XC StageTip and suspended in the loading buffer (0.5% TFA and 4% ACN) for LC/MS/MS analyses.

### *In vitro* Kinase Assay Using Cell Extracts

Cells were washed and harvested with ice-cold PBS. Proteins were extracted from HeLa cells with phase-transfer surfactant, and the buffer was replaced with 40 mM Tris-HCl (pH 7.5) by ultrafiltration using an Amicon Ultra 10K at 14,000*g* and 4 °C. Protein amount was determined with a BCA protein assay kit, and the solution was divided into aliquots containing 100 μg. As described above, proteins were dephosphorylated with TSAP, reacted with recombinant kinase, and digested with Lys-C (w/w 1:100) and trypsin (w/w 1:100), using the reported methods (14). Then, phosphopeptides were enriched from the tryptic peptides with HAMMOC, desalted using SDB-XC StageTips, and suspended in the loading buffer (0.5% TFA and 4% ACN) for LC/MS/MS analyses.

### NanoLC/MS/MS Analyses

NanoLC/MS/MS analyses were performed on an Orbitrap Fusion Lumos (Thermo Fisher Scientific) or on a Q Exactive (Thermo Fisher Scientific) for synthetic peptide samples, connected to an Ultimate 3000 pump (Thermo Fisher Scientific) and an HTC-PAL autosampler (CTC Analytics). Peptides were separated on a self-pulled needle column (150 mm length × 100 μm ID, 6 μm opening) packed with Reprosil-C18 AQ 3 μm reversed-phase material (Dr Maisch). The flow rate was set to 500 nl/min. The mobile phase consisted of (A) 0.5% acetic acid and (B) 0.5% acetic acid in 80% acetonitrile. Three-step linear gradients of 5–10% B in 5 min, 10–40% B in 60 min (for short gradient) or 100 min (for long gradient), and 40–100% B in 5 min were employed.

For BioID samples and phosphopeptides obtained by *in vitro* kinase reaction, the MS scan range was *m/z* 300–1500. MS scans were performed by the Orbitrap with *r* = 120,000 and subsequent MS/MS scans were performed by the Orbitrap with *r* = 1,5000. Auto gain control for MS was set to 4.00 × 10^5^ and that for MS/MS was set to 5.00 × 10^4^. The HCD was set to 30.

For TMT-labeled samples, synchronous precursor selection-MS3 (SPS-MS3) (36) was performed. The MS scan range was *m/z* 375–1500. MS scans were performed by the Orbitrap with *r* =120,000, MS/MS scans were performed by the Ion Trap in Turbo mode, and MS3 scans were performed by the Orbitrap with *r* = 1,5000. Auto gain control for MS was set to 4.00 × 10^5^, that for MS/MS was set to 1.00 × 10^4^, and that for MS3 was set to 5.00 × 10^4^. The CID was set to 35.

For synthetic peptide samples, the MS scan range was *m/z* 300–1500. MS scans were performed by the Orbitrap with *r* = 70,000, and subsequent MS/MS scans were performed by the Orbitrap with *r* = 17,500. Auto gain control for MS was set to 3.00 × 10^6^, and that for MS/MS was set to 1.00 × 10^5^. The HCD was set to 27.

### Database Searching

For all experiments, the raw MS data files were analyzed by MaxQuant v1.6.17.0 (37). Peptides and proteins were identified by means of automated database searching using Andromeda against the human SwissProt Database (version 2020-08, 20,368 protein entries) with a precursor mass tolerance of 20 ppm for first search and 4.5 ppm for main search and a fragment ion mass tolerance of 20 ppm. The enzyme was set as Trypsin/P with two missed cleavage allowed. Cysteine carbamidomethylation was set as a fixed modification. Methionine oxidation and acetylation on the protein N-terminus were set as variable modifications. For phosphopeptides, phosphorylations on serine, threonine, and tyrosine were also set as variable modifications. The search results were filtered with FDR <1% at the peptide spectrum match (PSM) and protein levels. Phosphosites were filtered to accept only those in which the localization score was >0.75.

### Data Analysis

For BioID samples, the peak area of each peptide in MS1 was quantified using MaxQuant. Missing values were imputed with values representing a normal distribution around the detection limit of the mass spectrometer. A two-tailed Welch's *t* test was performed comparing the BirA∗-kinase group to the control group. For the following analysis, the lead protein among the protein IDs was used.

For TMT samples, the peak intensities of reporter ions in MS3 were quantified using MaxQuant. Missing values were imputed with values representing a normal distribution around the detection limit of the mass spectrometer. The ratio of drug-treated to control was logged (base 2) for each phosphopeptide and averaged to each phosphosite. A two-tailed Welch's *t* test was performed comparing the BirA∗-kinase group with the control group. For the following analysis, the lead protein among the protein IDs was used.

For synthetic peptides, the peak area of each peptide in MS1 was quantified using MaxQuant and phosphorylation ratio was calculated as follows:

$$\text{Phosphorylation ratio }\left(\text{\%}\right)=\left(1-\frac{{\text{peak area}}_{\text{Nonphosphopeptide }\;(\text{w}/\text{kinase})}}{{\text{peak area}}_{\text{Nonphosphopeptide }\;(\text{w}/\text{o kinase})}}\right)\times 100$$

The protein–protein interaction (PPI) network analysis was performed with STRING version 11.0 (38) and the highest-confidence PPI network (score = 0.9) was used. GO analysis was performed with the Database for Annotation, Visualization, and Integrated Discovery (DAVID) version 6.8 (39, 40). The background was set as all proteins identified in the same measurement.

### Motif Score

The probability of observing residue x in position i from *in vitro* substrates for PKA or CK2 is computed as follows:

$$p\left(x,i\right)=\frac{f_{x,i}+c\left(x\right)}{N+\varepsilon }$$

c(x)=p(x)⋅ε

where $f_{x,i}$ is the frequency of observing residue x at position i, and N is the total number of sequences. c(x) is a pseudo count function, which is computed as the probability of observing residue b in the proteome, p(x), multiplied by ε, defined as the square root of the total number of sequences used to train the position weight matrix (PWM). This avoids infinite values when computing logarithms. Probabilities are then converted to weights as follows:

$$w_{x,i}=\log_{2}\frac{p\left(x,i\right)}{p\left(x\right)}$$

where p(x) = background probability of amino acid x; p(x,i) = corrected probability of amino acid x in position i; $W_{x,i}$ = PWM value of amino acid x in position i. Given a sequence q of length l, a score λ is then computed by summing log_2_ weights:

$$\text{λ}=\overset{l}{\underset{i=1}{\sum }}W_{q_{i},i}$$

where $q_{i}$ is the $i^{th}$ residue of q. In this study, the score was computed using the flanking ±7 residues surrounding each phosphosite.

## Results

### Defining Kinase-interacting Proteins Using BioID

**Fig. 1:**
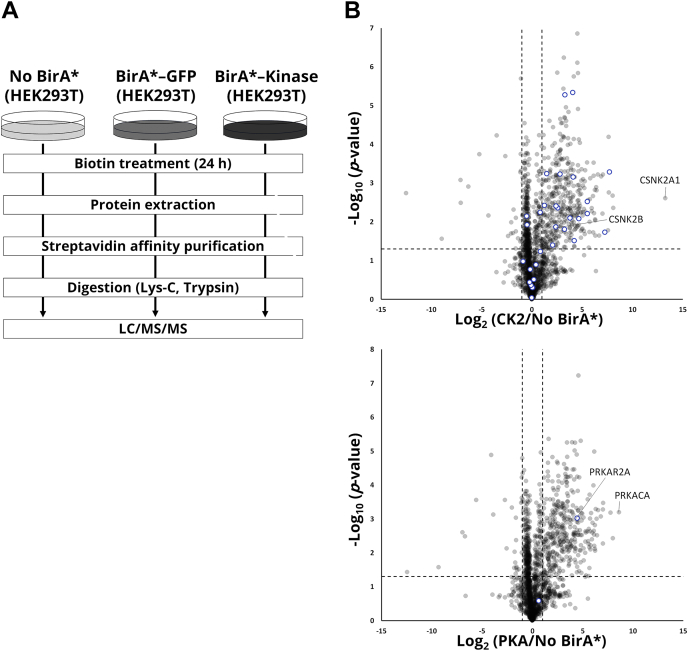
**Identification of kinase-interacting proteins with BioID.***A*, workflow to identify kinase-interacting proteins with BioID. *B*, the ratio of BirAk to control (log_2_ (BirAk/No BirA∗)) and the negative value of log_10_*p*-values (Welch's *t* test) are plotted for each protein. *Blue* indicates the known interactors of the given kinase (42).

To identify kinase-interacting proteins, we first performed BioID experiments (Fig. 1*A*). We selected CK2 and PKA as target kinases, because their functions, localizations, and putative substrates have been relatively well investigated (8). BirA∗-fused kinases (BirAk) were individually transfected into HEK293T cells, and BirA∗-fused GFP (BirAg) transfected or nontransfected (No BirA∗) cells were used as a negative control. For the labeling time of biotin, we set a value of 24 h, which has been adopted in many previous studies (30, 32). We observed a broad spectrum of biotinylated proteins by streptavidin blotting for the BirAk or BirAg-expressing cells, while no signals were detected for the nontransfected control cells (supplemental Fig. S1*A*). Furthermore, we conducted *in vitro* kinase reaction using synthetic peptides with immunoprecipitated BirAk or recombinant kinase. The intensity values of phosphorylated peptides between the immunoprecipitated BirAk and the recombinant kinase were highly correlated, indicating that the BirAks expressed in cells were active (supplemental Fig. S1*B*). These results confirm the validity of our BioID experiments. After the transfection and biotin addition, biotinylated proteins were enriched with streptavidin beads and digested to obtain tryptic peptides, which were analyzed by nanoLC/MS/MS and quantified using label-free quantification (41). As a result, 1713 proteins and 1678 proteins were quantified in at least two of the three replicates in the CK2- and PKA-expressing cells, respectively. To select a proper control for our BioID experiments, we evaluated two controls. As illustrated in Figure 1*B* and supplemental Figure S1, *C* and *D*, quantified proteins were mapped on volcano plots based on the significance and the ratio between BirAk and controls. For BirA∗-CK2, the known interactors showed higher ratios of BirAk/control with both controls, whereas for BirA∗-PKA, the BirA∗-GFP control generated false-negatives such as the well-known interactor PRKAR2A. A similar tendency was observed for known substrates. Based on these results, we adopted nontransfected cells (No BirA∗) as the control for the following analysis.

Among them, 407 and 370 proteins were considered as interacting proteins of CK2 and PKA, respectively, after applying the cutoff values (supplemental Table S1). Importantly, CSNK2B and PRKAR2A, which form heteromeric complexes with the corresponding kinases, were significantly enriched as kinase interactors (Fig. 1*B*), suggesting that the tagged kinases form the heteromeric complexes *in vivo* and are likely to function in the same manner as the endogenous kinases.

To benchmark our method, we mapped CK2- or PKA-interacting proteins (n = 407 and n = 370, respectively) on the STRING (38)-based protein interaction networks. We found that 265 proteins (corresponding to 67%, *p*-value < 10^−16^) and 230 proteins (63%, *p*-value < 10^−16^) formed single large networks (that is, any given pair of proteins is connected directly and/or indirectly) (supplemental Fig. S1*E*). This indicates that our BioID experiments capture biologically meaningful proteins proximal to the target kinases. Finally, we performed gene ontology (GO) enrichment analyses for the sets of kinase-interacting proteins (supplemental Table S2). We found that GO terms related to "RNA processing" and "apoptosis" were enriched in the CK2-interacting proteins, consistent with the known functionality and biology of the CK2 family (42, 43). For the PKA-interacting proteins, we observed the term "cell-cell adhesion" in line with PKA's known function (44).

Altogether, the obtained results suggest that our data have a high level of confidence and represent a rich source of CK2 and PKA interactomes including both stable and transient interactors.

### Phosphoproteomic Profiling of Responses to Kinase Perturbations

**Fig. 2:**
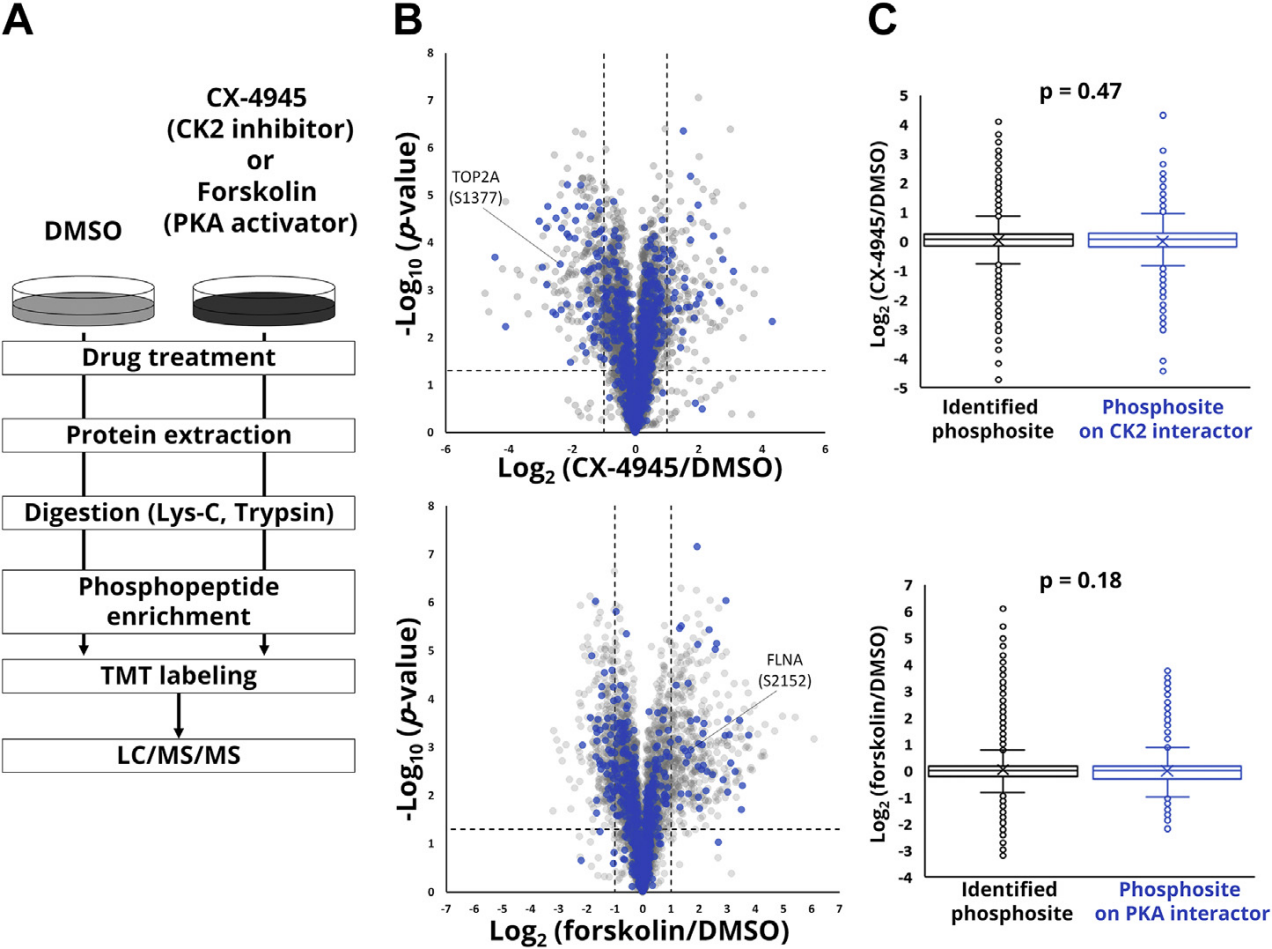
**Identification of phosphosites regulated by the target kinase**. *A*, quantitative phosphoproteomics to profile phosphorylation changes in the presence of target kinase inhibitor/activator. *B*, Volcano plot for the log_2_ ratios between the CX-4945-treated or forskolin-treated and dimethylsulfoxide-treated HEK293T cells in biological triplicates. *Each dot* represents a phosphosite. *Blue color* indicates sites on the protein interacting with the target kinase. *C*, Boxplot of phosphorylation ratio of all identified phosphosites and phosphosites on proteins interacting with the target kinase. x indicates the average value. *p*-value was calculated using Welch's *t* test.

Having established the CK2 and PKA interactomes, we next sought to perform kinase-perturbed phosphoproteome profiling to identify phosphosites regulated by the target kinases (Fig. 2*A*). To this end, HEK293T cells were treated with either an ATP-competitive CK2 inhibitor, CX-4945, also known as silmitasertib (45), or a PKA activator, forskolin (46). Following protein extraction and tryptic digestion, phosphopeptides were enriched by means of titania chromatography (35), labeled with TMT reagents and analyzed by nanoLC/MS/MS. To simplify the interpretation of kinase–phosphosite relationships, we only considered the quantitative results of monophosphopeptides in subsequent analyses; as a result, 7188 and 7087 monophosphosites were quantified in triplicate from CX-4945- and forskolin-treated cells, respectively (supplemental Table S3). Downregulation of known CK2 substrates such as TOP2A (S1377) and LIG1 (S66) and upregulation of known PKA substrates such as STMN1 (S16), NDE1 (S306), and FLNA (S2152) were confirmed, supporting the validity of our results (Fig. 2*B* and supplemental Table S3). Furthermore, expected sequence motifs for acidophilic CK2 and basophilic PKA were clearly enriched in perturbed phosphosites (2-fold or more upon CK2 inhibition as an average of biological triplicates and with a *p*-value less than 0.05, and upregulated by 2-fold or more upon PKA activation as an average of biological triplicates and with a *p*-value less than 0.05) (supplemental Fig. S2), demonstrating that the data obtained in this experiment are meaningful and of high quality.

To combine the results obtained by the kinase-perturbed phosphoproteomics with those in the BioID experiments, we mapped the identified phosphorylation sites onto the kinase-interacting proteins; 981 and 772 phosphosites were attributed to the 265 and 230 proteins interacting with CK2 and PKA, respectively (Fig. 2*B*). There was no significant correlation between the kinase interactor proteins and the phosphosites perturbed by the kinase modulators (Fig. 2*C*), indicating that these orthogonal methods can identify endogenous substrates with high confidence. We then set the cutoff, 2-fold change on average (n = 3) with *p*-value < 0.05, to further select the regulated phosphosites, resulting in the identification of 62 and 44 phosphosites for CK2 and PKA substrate candidates, respectively.

### Phosphorylation Motif Analysis

Our kinase-perturbed phosphoproteome analysis identified both directly and indirectly regulated sites due to the perturbation of downstream kinases of the target kinases or off-target effects of the drugs used for perturbation. Thus, we next selected phosphosites that match the consensus phosphorylation motif of the kinase, which has been shown to be a useful predictor in kinase identification (21).

**Fig. 3:**
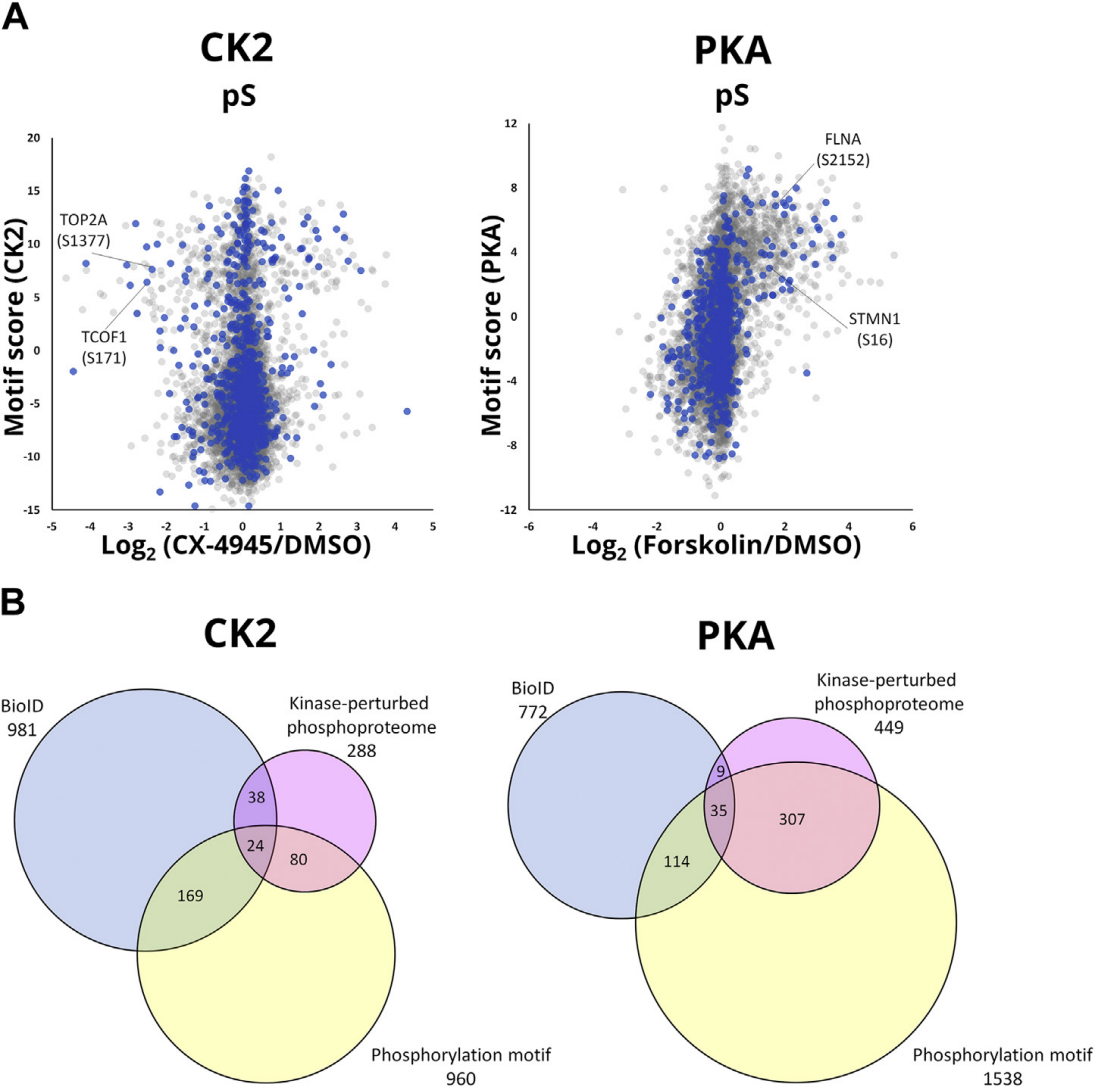
**Identification of phosphosites phosphorylated by the target kinase.***A*, the distribution of phosphorylation ratios and motif scores for phosphosites identified in cells treated with inhibitor or activator. *Blue color* indicates sites on the protein interacting with the target kinase. *B*, Venn diagram showing the overlap of the phosphosites that passed through the three filters.

PWM (47) represents the signatures of the sequences flanking the phosphosites targeted by a given kinase (supplemental Fig. S3, *A* and *B*). Here columns in the matrix represent relative positions from phosphosites, while rows represent residues. The values in the matrix are log2-transformed probabilities of a residue's occurrence at a position. These probabilities are used to predict putative substrates (see methods). This approach has been implemented in several kinase–substrate prediction methods and applied to *in vivo* kinase−substrate discovery. We built the PWM based on thousands of *in vitro* substrates identified in our previous study (14) as well as in this study (Fig. 3*A*, supplemental Fig. S3, *A* and *B*) and calculated the motif scores for the substrate candidates. As illustrated in Figure 3*A* and supplemental Fig. S3*C*, some phosphosites showed low motif scores, although they passed the criteria set for BioID and kinase-perturbed phosphoproteomics. This result indicates that phosphorylation motif analysis is essential and orthogonal to both the kinase interactome and the kinase-perturbed phosphoproteome. Furthermore, due to the large number of *in vitro* substrates used as the training set for PWM, several phosphosites were rescued, especially for PKA, that would have been rejected if the number of *in vitro* substrates had been limited (supplemnatl Fig. S3*D*). This means that the number of *in vitro* substrates used in the PWM training set is important to distinguish true substrates. Finally, 24 CK2 substrates and 35 PKA substrates met the criteria (motif score >2, supplemental Table S3, Fig. 3*B*). They include well-known substrates such as TOP2A (S1377) for CK2 and FLNA (S2152) and STMN1 (S16) for PKA. Notably, such well-known substrates were not identified by conventional AP-MS (48) possibly due to the transient and unstable interaction. Note that only five substrate proteins (CDC5L, CEP170, SAP30BP, TCOF1, THRAP3) and none of our high-confidence substrate proteins are described as interactors of CK2 and PKA, respectively (48), meaning that other substrate proteins may be transient and unstable interactors that can only be captured by BioID. The GO enrichment analysis revealed that RNA splicing-related proteins were enriched as CK2 substrates, which is consistent with CK2 being an RNA splicing-related kinase (49), while cell adhesion-related molecules were identified as PKA substrates, in accordance with PKA's involvement in the cell–cell adhesion pathway (44).

### Missense Mutations Near Phosphosites of CK2 and PKA Substrates

**Fig. 4:**
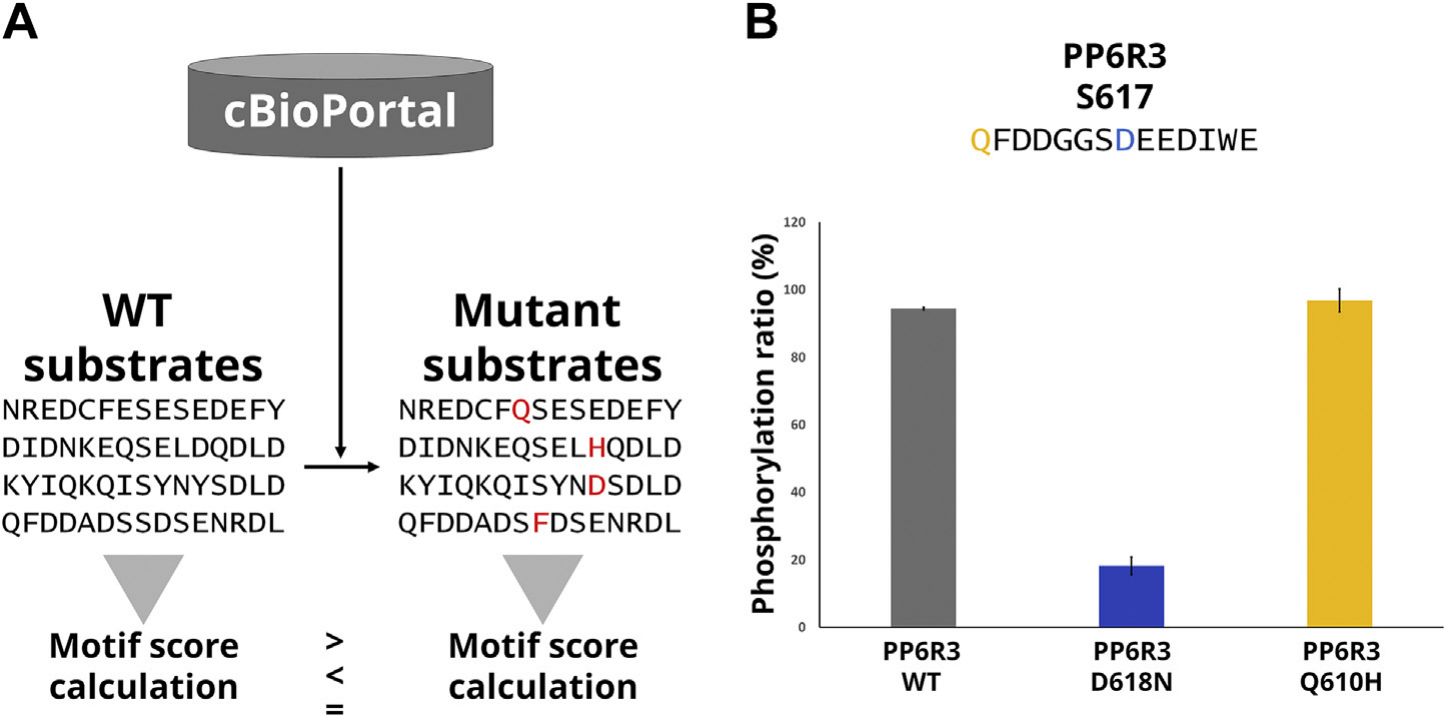
***In vitro* kinase assay of putative kinase substrates using synthetic peptides**. *A*, workflow to evaluate the effect of amino acid substitutions in substrates. *B*, PP6R3 (S617).

Having discovered putative direct substrates of CK2 and PKA, we next sought to assess the relationship between the kinases and substrates in the context of cancer biology (50, 51). The effect of amino acid substitutions in substrates on the kinase–substrate relationship has been analyzed using kinase substrate sequence specificity (52, 53). Information on amino acid substitutions that occur within seven residues around the phosphosites in supplemental Table S3 was extracted from the cancer genomics database cBioPortal (54, 55) (Fig. 4*A*). As a result, 80 missense mutations from 24 CK2 substrates and 139 missense mutations from 35 PKA substrates were found. For substrate sequences containing these mutations, we used the motif scores to predict phosphorylation preference by kinases and compared them with the wild-type sequences (supplemental Fig. S4). Then, wild-type/mutant substrate pairs of PP6R3 for CK2 that showed potential loss or no change of phosphorylation by mutation were selected for *in vitro* kinase assays using synthetic peptides (Fig. 4*B*). As a result, mutation-induced reduction in phosphorylation stoichiometry was observed in the mutations that were predicted to decrease the activity, whereas phosphorylation stoichiometry was not changed in the mutations that were predicted not to change the activity. These results indicate that missense mutations near phosphosites of substrates negatively affect phosphorylation by CK2 or PKA and thus would be expected to affect downstream signaling networks in cancer cells bearing these mutations.

## Discussion

Knowledge of kinase–substrate relationships is critical to understand intracellular signaling networks. We believe the present study is the first to identify endogenous kinase substrates by combining three approaches: proximity labeling, kinase-perturbed phosphoproteomics, and phosphorylation motif analysis. This strategy enabled us to identify novel endogenous substrates as well as known endogenous substrates and offers many advantages. First, BioID is able to identify transient and unstable interactors that could not be found by conventional AP-MS methods. Second, phosphorylation motif analysis can be applied to any sequence, as it scores matches against substrate motifs rather than against substrates obtained in *in vitro* kinase assay. Finally, this combined approach should be generally applicable to any kinase.

Using BioID, we identified novel interactor candidates not picked up by AP-MS data, and these new interactor candidates led to 17 new CK2 substrates and 35 new PKA substrates (48). In general, AP-MS cannot capture the substrate proteins, because the phosphorylated product is immediately released by the kinase (27). Therefore, we thought proximity labeling would be the best approach to search for endogenous kinase substrates. Public PPI databases such as BIOGRID are a potential resource for probing the kinase interactome (56). However, the data in BIOGRID was obtained with conventional methods, such as AP-MS and yeast two-hybrid studies, and therefore may be a poor source of kinase interactome data. Indeed, we identified many interactors that were not picked up by the conventional methods, leading to the identification of ten new CK2 substrates and 34 new PKA substrates. Thus, since public PPI databases may not be effective tools to identify true kinase substrates, we employed BioID experiments to identify kinase interactors, using the same cells as in other experiments for convenience.

There are a variety of proximity-dependent biotinylation approaches, such as APEX and TurboID (57). APEX is a powerful tool in terms of labeling speed, but requires the use of H_2_O_2_, which is cytotoxic. TurboID catalyzes biotinylation faster than BioID and was considered more suitable for ourpurpose. There has been extensive discussion about what kind of control samples should be used in BioID experiments (57). Here, we prepared two different controls and compared them. We found that the BirA∗-GFP control failed to identify true interactors, while the No BirA∗ control resulted in more nonsubstrate proximal proteins. Therefore, additional filters that should be independent and orthogonal to the kinase interactome are required. We found that kinase-perturbed phosphoproteome analysis and phosphorylation motif analysis were effective to minimize nonsubstrate proximal proteins in the kinase interactome analysis.

In this study, we employed well-known specific kinase inhibitors or activators to obtain kinase-perturbed phosphoproteome profiles (58, 59). Knockdown, knockout, or overexpression of the gene of target kinase would be alternative approaches, especially in the case of kinases for which no specific inhibitor or activator is available, but these methods change the proteome profile of the cell, as well as the expression level of the target kinase (20).

Phosphorylation motif analysis is an important step in our strategy. We found that the motif score depends on the size of the training data set, with a smaller size reducing the quality as a filter for identifying kinase substrates. However, simply matching whether a phosphosite has a consensus motif is considered to be too loose as a filter. To evaluate our identified substrates, we compared them with known substrates in a public database, PhosphoSitePlus (4). Only three substrates overlapped between PhosphoSitePlus and our results. Many of the known substrates were not quantified in our BioID experiment or in our kinase-perturbed phosphoproteome, possibly due to the random sampling in LC/MS/MS or low abundance in HEK293T cells. In addition, the relationships between upstream kinases and substrates listed on PhosphoSitePlus are not necessarily those that hold under physiological conditions. In fact, some substrates in PhosphoSitePlus were identified but dropped by one of our three filters. Nevertheless, as there is still no way to identify physiological kinase substrates with high throughput, we believe that our method represents a step toward overcoming that issue.

One of the likely applications of this approach is the identification of endogenous kinase substrate mutations associated with cancer. For example, our present analysis demonstrates the potential impact of known oncogenic mutations on phosphorylation-related signaling pathways and illustrates the usefulness of motif analysis for screening. We believe the methodology described here will be useful for large-scale discovery of endogenous kinase substrates.

## Data availability

The mass spectrometry proteomics data have been deposited at the ProteomeXchange Consortium (http://proteomecentral.proteomexchange.org) *via* the jPOST partner repository (http://jpostdb.org) (60) with the data set identifier PXD019664.

## Supplemental data

This article contains supplemental data (61).

## Conflict of interest

Authors declare no competing interests.
