# Supplementary material for: Identification of Endogenous Kinase Substrates by Proximity Labeling Combined with Kinase Perturbation and Phosphorylation Motifs
Source: Mol Cell Proteomics. 2021 Jun 27;20:100119. doi: 10.1016/j.mcpro.2021.100119 (PMC8325102; doi:10.1016/j.mcpro.2021.100119)
Supplement: Supplemental Figures S1–S4 [file mmc1.docx]

**Supplemental Data**

**Identification of Endogenous Kinase Substrates by Proximity Labeling Combined with Kinase Perturbation and Phosphorylation Motifs**

Tomoya Niinae^1^, Koshi Imami^1, 2^, Naoyuki Sugiyama^1^ and Yasushi Ishihama^1, 3^*

1) Department of Molecular & Cellular BioAnalysis, Graduate School of Pharmaceutical Sciences, Kyoto University, 46–29 Yoshidashimoadachi-cho, Sakyo-ku, Kyoto 606–8501, Japan

2) PRESTO, Japan Science and Technology Agency (JST), 5-3 Yonban-cho, Chiyoda-ku, Tokyo, 102-0075, Japan

3) Laboratory of Clinical and Analytical Chemistry, National Institute of Biomedical Innovation, Health and Nutrition, Ibaraki, Osaka, 567-0085, Japan.

**Corresponding author:** Yasushi Ishihama (yishiham@pharm.kyoto-u.ac.jp)

**Table of Contents**

Figure S1 BioID for identification of CK2 and PKA interactors.

Figure S2 Kinase-perturbed phosphoproteome analysis.

Figure S3 Position weight matrix of CK2 and PKA substrates.

Figure S4 Comparison of predicted phosphorylation of WT and mutant substrates based on motif scores.

Table S1 CK2 and PKA interactors identified in the BioID experiments.

1. Identified peptides from CK2 interactors
2. Identified peptides from PKA interactors
3. Identified proteins as CK2 interactors
4. Identified proteins as PKA interactors

Table S2 GO enrichment analysis of identified CK2 and PKA interactors in the BioID experiments.

(A) GO biological process of identified CK2 interactors

(B) GO cellular component of identified CK2 interactors

(C) GO biological process of identified PKA interactors

(D) GO cellular component of identified PKA interactors

Table S3 Phosphosites identified in kinase-perturbed phosphoproteome analysis.

1. Identified phosphopeptides from CX-4945 treated cells
2. Identified phosphopeptides from forskolin treated cells
3. Identified phosphosites from CX-4945 treated cells
4. Identified phosphosites from forskolin treated cells


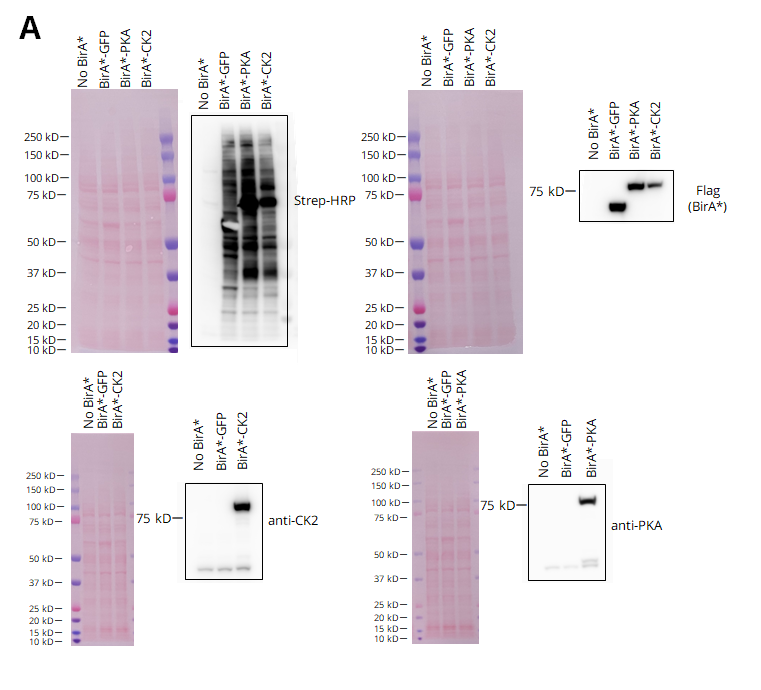

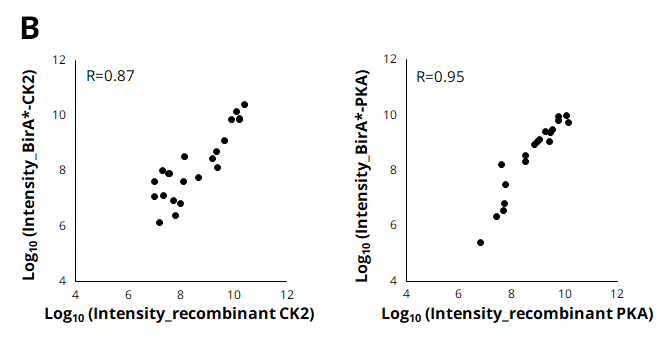

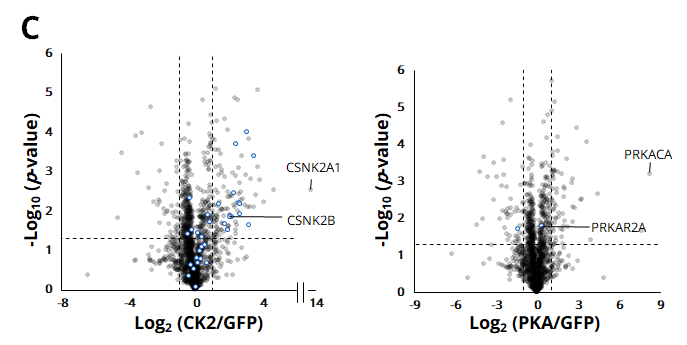

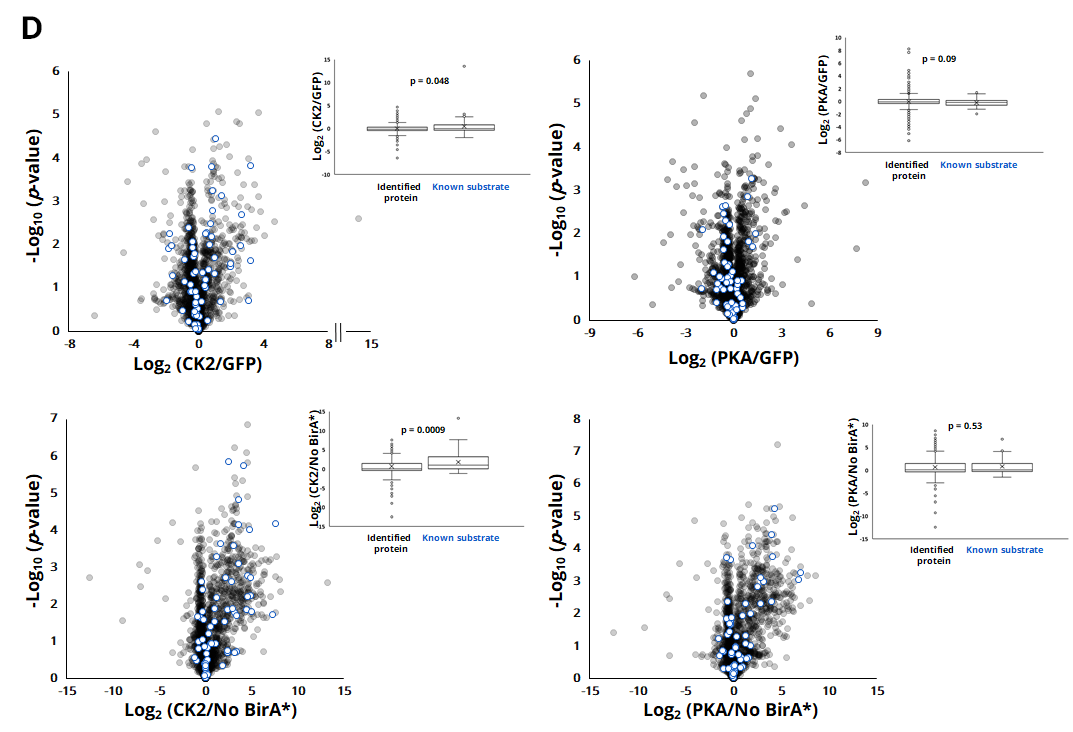

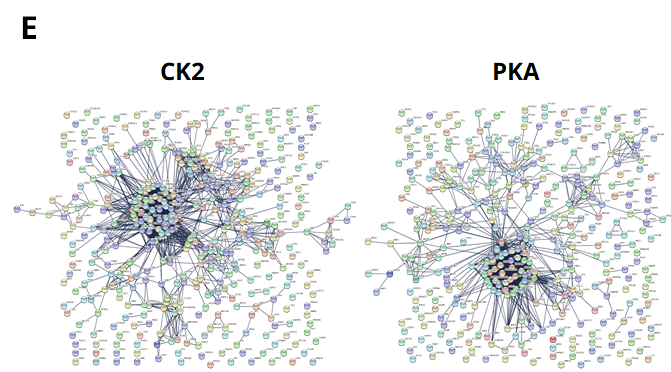


**Figure S1. BioID for identification of CK2 and PKA interactors.**

(A) Validation of biotinylation by western blotting and expression of BirAk.

(B) Validation of the kinase activity of BirAk. Each dot represents a phosphorylated peptide. X axis is the intensity of peptides phosphorylated by immunoprecipitated BirAk minus the intensity of the corresponding peptides in the control (BirAg). Y axis is the intensity of peptides phosphorylated by recombinant kinase.

(C) The ratio of BirAk to control (log_2_ (BirAk/BirAg)) and the negative value of log_10_ *p*-values (Welch’s t-test) are plotted for each protein. Blue indicates the known interactors of the given kinase.

(D) The ratio of BirAk to control (log_2_ (BirAk/BirAg or No BirA*)) and the negative value of log_10_ *p*-values (Welch’s t-test) are plotted for each protein. Blue indicates the known substrates of the given kinase.

(E) PPI network of interacting proteins. PPI network analysis was performed with STRING and the highest-confidence PPI network (score = 0.9) was used.


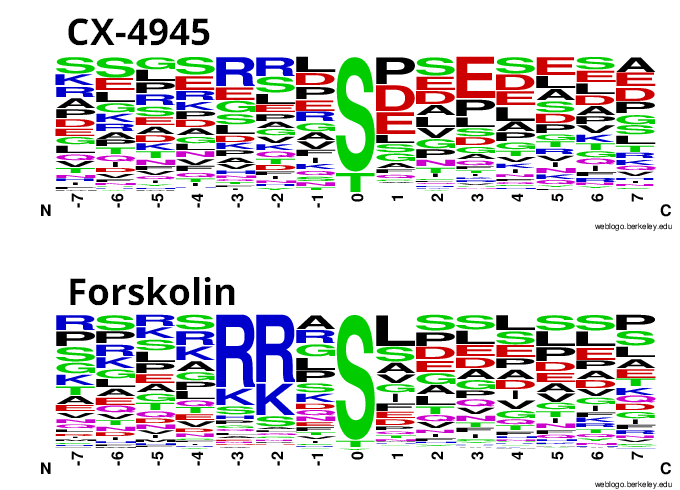


**Figure S2. Kinase-perturbed phosphoproteome analysis.**

Motifs down-regulated by 2-fold or more upon CK2 inhibition as an average of biological triplicates and with a p-value less than 0.05, and up-regulated by 2-fold or more upon PKA activation as an average of biological triplicates and with a p-value less than 0.05. Frequency plots were visualized by WebLogo (v. 2.8.2) (61).


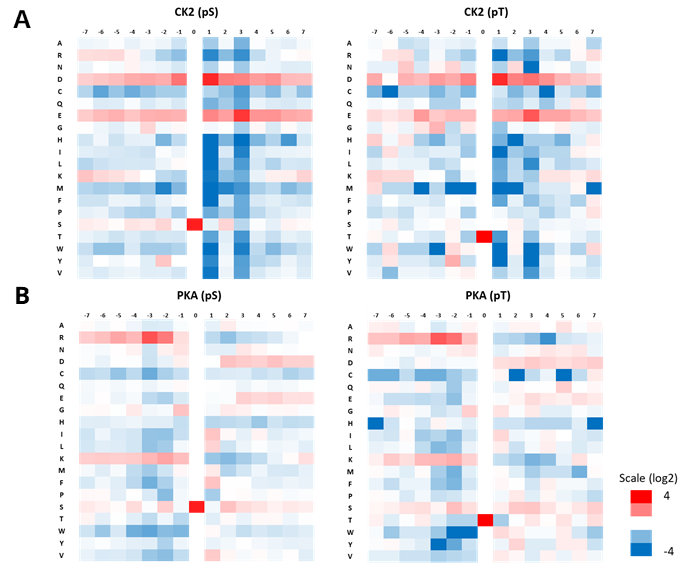

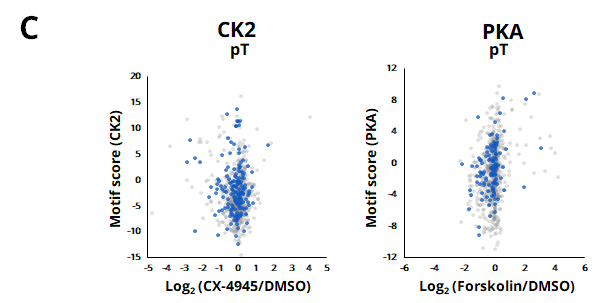

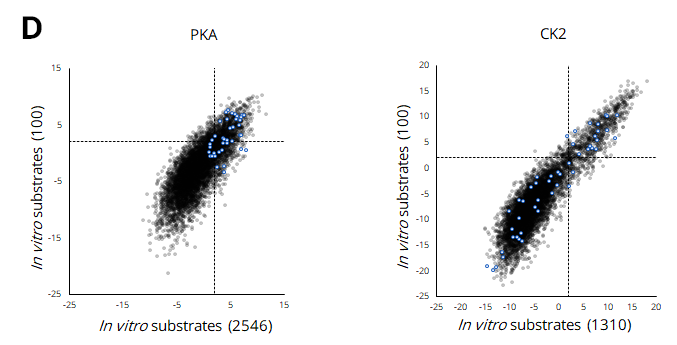


**Figure S3. Position weight matrix of CK2 and PKA substrates.**

Columns in the matrix represent relative positions from phosphosites. Rows represents residues. The values in the matrix are log2-transformed probabilities of a residue’s occurrence at a position. (A) CK2 (B) PKA

(C) The distribution of phosphorylation ratios and motif scores for phosphosites (pT) identified in cells treated with inhibitor or activator. Blue color indicates sites on the protein interacting with the target kinase.

(D) Comparison of motif scores computed based on different training dataset sizes. Blue indicates the phosphosites that passed through BioID and kinase-perturbed phosphoproteome. The number in parentheses in the axis name is the number of substrates in the training set.

**
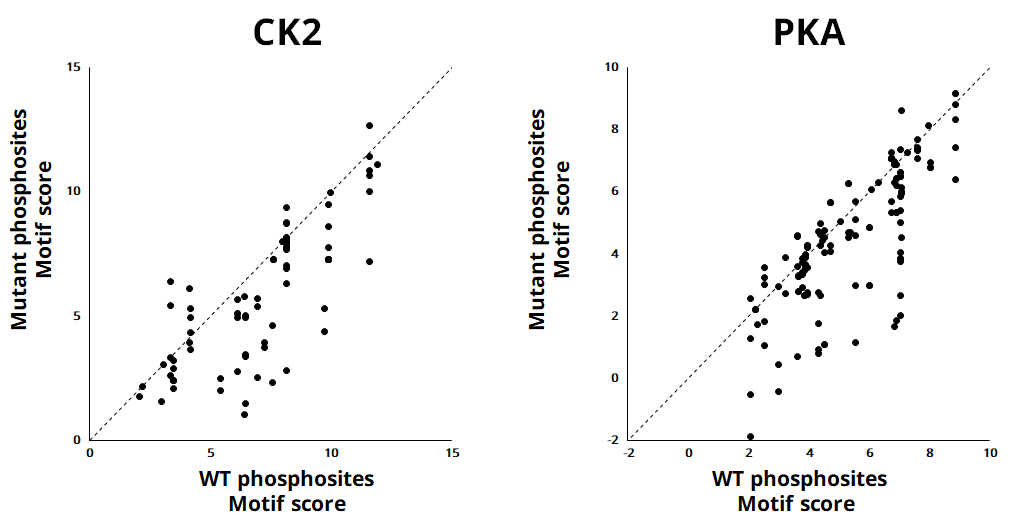
**

**Figure S4. Comparison of predicted phosphorylation of WT and mutant substrates based on motif scores.**

Comparison of motif scores between WT and mutant sequence. (A) CK2 (B) PKA

**References**

61.     [Crooks, G. E. (2004) WebLogo: A Sequence Logo Generator. *Genome Research* 14, 1188–1190](http://paperpile.com/b/XSI5wO/VVUA)
